# History of a Broncholith, Bronchial Calculus, or Lung Stone

**Published:** 1913-10

**Authors:** Walter F. Chappell

**Affiliations:** New York


### History of a Broncholith, Bronchial Calculus, or Lung Stone.

Dr. Walter F. Chappell, New York: This case is cited to show that all causes of asthma are not centered in the nose, nor all throat coughs in the larynx. The patient was a woman, 52 years of age, who was attacked by severe wheezing, the spasmodic cough lasting an hour at a time, and by intense tickling in the throat. The tickling was below the larynx, and the wheezing in the upper part of the chest, in front and behind. The patient soon raised a small calcareous mass, but the asthma continued. Another coughing spell resulted in the expulsion of a large calcareous mass. Immeliately all wheezing stopped and the breathing became absolutely free. Later another calcareous mass was coughed up. Chemically these masses consisted of phosphate of lime. The patient gave no history that would give a clue to the cause for the existence of this condition.

